# Five new Lamiinae (Coleoptera, Cerambycidae) from Bolivia in honor of James E. Wappes

**DOI:** 10.3897/zookeys.488.9060

**Published:** 2015-03-19

**Authors:** Maria Helena M. Galileo, Ubirajara R. Martins, Antonio Santos-Silva

**Affiliations:** 1 PPG Biologia Animal, Departamento de Zoologia, Universidade Federal do Rio Grande do Sul, Porto Alegre, RS, Brazil (researcher of CNPq); 2 Museu de Zoologia, Universidade de São Paulo, São Paulo, SP, Brazil (researcher of CNPq); 3 Museu de Zoologia, Universidade de São Paulo, São Paulo, SP, Brazil

**Keywords:** Keys, South America, Taxonomy

## Abstract

Five new species of Lamiinae are described from Bolivia, all named after James E. Wappes: *Xenofrea wappesi* (Xenofreini); *Anobrium wappesi* (Pteropliini); *Cotycicuiara wappesi*, *Nesozineus wappesi*, and *Psapharochrus wappesi* (Acanthoderini). *Anobrium wappesi*, *Cotycicuiara wappesi*, and *Nesozineus wappesi* are included in known keys. A short note on the name and date of *Anobrium oberthueri* Belon, 1903 is provided.

## Introduction

During recent years, particularly in the last ten years, James E. Wappes has sent a large number of longhorn beetles to the MZSP (see below) for identification. Most of the specimens have come from Bolivia, where he has concentrated his collection efforts. New species have often been found and named after James. In this work, we describe five new species which are named after him. Thus, the species described have the same etymology.

The Cerambycidae fauna of Bolivia (Monné 2014, cat.) continues to surprise researchers with incredible numbers of new species found there. The five new species of Cerambycidae described in this work are only a small portion of the species from Bolivia that remain undescribed.

## Materials and methods

Photographs were taken with Canon EOS Rebel T3i DSLR camera, Canon MP-E 65mm f/2.8 1–5X macro lens, controlled by Zerene Stacker AutoMontage software.

The acronyms used in the text are as follows:

ACMT American Coleoptera Museum (James E. Wappes), San Antonio, Texas, USA.

MNKM Museo de Historia Natural Noel Kempff Mercado, Santa Cruz, Bolivia.

MZSP Museu de Zoologia, Universidade de São Paulo, São Paulo, Brazil.

## Taxonomy

### Xenofreini Aurivillius, 1923

#### Xenofrea wappesi sp. n.

Taxon classificationAnimaliaColeopteraCerambycidae

http://zoobank.org/74C4E719-FABE-4FB3-8CD1-EF49985A98C0

Figs 1–3

##### Description.

*Holotype male.* Color. Integument dark brown, almost black, except for scape, distal third of pedicel, distal quarter of antennomeres III–IV, basal two-thirds of antennomeres V–VII, basal third of antennomere VIII, most of gulamentum, most of peduncle of meso- and metafemora, and basal half of tibiae which are brown; basal three-quarters of antennomeres III–IV reddish-brown.

*Head.* Frons trapezoidal; finely, densely punctate, interspersed with coarse, sparse punctures; with white pubescence, not completely concealing integument. Area between antennal tubercles and upper eye lobes concave; moderately coarsely, abundantly punctate; pubescence white, mixed with yellowish white pubescence. Antennal tubercles finely, abundantly punctate; with white pubescence mixed with yellowish white pubescence. Dorsal area between eyes and anterior edge of prothorax coarsely punctate; pubescence very sparse, slightly more conspicuous around coronal suture. Coronal suture distinct from clypeus to anterior edge of prothorax. Area behind eyes with white pubescence (more yellowish depending on angle of incidence of light). Genae with yellowish white pubescence, sparser under lower eye lobe. Distance between upper eye lobes, in frontal view, equal to 0.45 times length of scape; distance between lower eye lobes equal to 0.80 times length of scape. Antennae as long as 1.7 times elytral length, reaching elytral apex at apex of antennomere VIII; scape, pedicel and lighter areas of antennomeres III–VIII with white pubescence, not obliterating integument; antennal formula based on antennomere III: scape = 0.59; pedicel = 0.22; IV = 0.94; V = 0.59; VI = 0.54; VII = 0.50; VIII = 0.44; IX = 0.42; X = 0.37; XI = 0.35.

**Figures 1–6.**
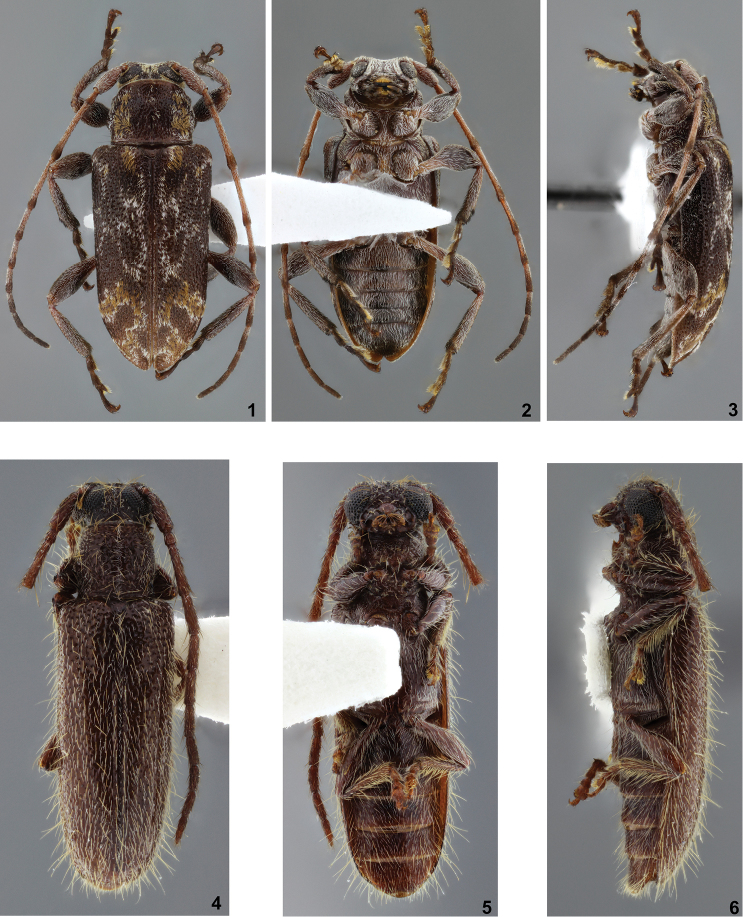
**1–3** *Xenofrea wappesi*, holotype male: **1** dorsal habitus **2** ventral habitus **3** lateral habitus **4–6** *Anobrium wappesi*, holotype female: **4** dorsal habitus **5** ventral habitus **6** lateral habitus.

*Thorax.* Prothorax transverse, largest width 1.4 times central length. Pronotum, moderately finely, abundantly punctate; anterior and posterior transverse sulcus wide, moderately deep; central pubescence white, very sparse; wide lateral band of yellowish brown pubescence mixed with white, not reaching anterior and posterior margin, adjacent to wide band of white pubescence. Lateral sides of prothorax with punctures denser than on pronotum; with long, sparse, dark setae on basal half; pubescence whitish, distinctly not concealing integument. Ventral surface with whitish pubescence, not obliterating integument; on metasternum, in front of mesocoxal cavities and mesosternal process, narrow band of white, dense pubescence. Scutellum with sparse white and yellowish brown pubescence. Elytra. Moderately coarsely, abundantly punctate; elongate, with lateral sides sub-parallel at basal two-thirds; apex narrow, individually rounded; short, elongate band of yellowish brown pubescence on each side of scutellum, internally margined with white; above humeri, small spot of yellowish brown pubescence; remaining surface of basal two-thirds with white pubescence, forming designs (Fig. 1), but mostly glabrous; distal third with sinuous, wide band of yellowish brown pubescence, margined with white pubescence (Fig. 1).

*Abdomen.* Urosternites finely, densely punctate; pubescence whitish, not obliterating integument; on urosternite I, between metacoxal cavities, narrow, dense, V-shaped band of white pubescence. Legs. Femora and tibiae with white, not dense pubescence. Club of metafemora not notably enlarged.

*Dimensions in mm* (male/female). Total length, 6.05/7.05; length of prothorax at center, 1.30/1.60; anterior width of prothorax, 1.50/1.90; posterior width of prothorax, 1.55/1.80; humeral width, 2.25/2.75; elytral length, 4.55/5.25.

*Variability.* Paratype female. Peduncle of meso- and metafemora dark-brown. Antennae as long as 1.6 times elytral length; reaching elytral apex at apex of antennomere IX.

##### Type material.

Holotype male from BOLIVIA, *Santa Cruz*: Florida (4 km N Bermejo; Refugio los Volcanes; 18°06'S / 63°36'W; 1045–1350 m), 4–9.XII.2013, Wappes & Skillman col. (MNKM). Paratype female, same data as holotype, except for: (1000–1200 m), 29.X.2011, Skillman & Wappes col. (ACMT).

##### Remarks.

*Xenofrea wappesi* differs from *Xenofrea areolata* Bates, 1885, *Xenofrea ocellata* Tavakilian & Néouze, 2006, and *Xenofrea punctata* Galileo & Martins, 2005 as follows: body distinctly narrower; central area of pronotum with sparse, white pubescence; basal two-thirds of elytra mostly with white, narrow bands of pubescence. In *Xenofrea areolata* and *Xenofrea punctata* the body is wider (mainly in *Xenofrea areolata*), the central area of pronotum has a band of orange pubescence, and the basal two-thirds of elytra has wide bands of orange pubescence. *Xenofrea wappesi* also differs from *Xenofrea punctata* and *Xenofrea ocellata* by the punctures on elytra distinctly smaller. It can be distinguished from *Xenofrea basitriangularis* Néouze & Tavakilian, 2005, *Xenofrea berkovae* Néouze & Tavakilian, 2005, *Xenofrea morvanae* Néouze & Tavakilian, 2005, and *Xenofrea murina* Néouze & Tavakilian, 2005 mainly by the antennae, which are distinctly shorter (in males, surpassing the elytral apex by two segments). In males of *Xenofrea basitriangularis*, *Xenofrea berkovae*, *Xenofrea morvanae* and *Xenofrea murina*, the antennae surpass the elytral apex by more than three segments.

### Pteropliini Thomson, 1860

#### Anobrium wappesi sp. n.

Taxon classificationAnimaliaColeopteraCerambycidae

http://zoobank.org/E5546F1D-64CB-4BD7-835A-E7788F1E1943

Figs 4–6

##### Description.

*Holotype female.* Color. Integument dark-brown; scape, pedicel, antennomeres III–V, and tarsi brown.

*Head.* Frons coarsely, abundantly punctate; with short, sparse, white setae, interspersed with long, sparse, yellowish setae. Area between antennal tubercles and posterior edge of upper eye lobes coarsely punctate (punctures confluent towards frons); setae as on frons. Area between upper eye lobes and anterior edge of prothorax finely, sparsely punctate, with very short, sparse white setae. Coronal suture distinct from clypeus to anterior edge of prothorax. Area behind lower eye lobes microsculptured, with long, sparse, yellowish setae close to eye. Distance between upper eye lobes, in frontal view, equal to 0.35 times length of scape; distance between lower eye lobes equal to 0.70 times length of scape. Antennae as long as 1.25 times elytral length; reaching distal sixth of elytra; scape and pedicel with short, sparse white setae, interspersed with long, yellowish setae, mainly ventrally; antennomeres III–VII with short, sparse white setae, interspersed with long, yellowish and brownish setae; antennomeres VIII–XI with short brownish setae, interspersed with long brownish setae; antennal formula based on antennomere III: scape = 0.85; pedicel = 0.26; IV = 0.79; V = 0.70; VI = 0.68; VII = 0.62; VIII = 0.56; IX = 0.53; X = 0.53; XI = 0.56.

*Thorax.* Prothorax cylindrical, approximately equally long as wide; lateral sides slightly rounded, without tubercles. Pronotum coarsely, moderately abundantly punctate; centrally, from base to apex, with band of white, short setae, with wide band of very sparse, white setae on each side; laterally with short, moderately abundant white setae; whole surface with long, sparse, yellowish setae. Lateral sides of prothorax with moderately sparse, white setae interspersed with long, sparse yellowish setae. Pro- and mesosternum with short, sparse, white setae interspersed with long, sparse yellowish setae. Metasternum with moderately abundant, white setae, interspersed with long, sparse, white and yellowish setae. Elytra. Moderately coarsely, abundantly punctate (punctures finer, sparser towards apex); with moderately abundant, white setae interspersed with long, yellowish setae; apices together rounded.

*Abdomen.* Urosternites shallowly, sparsely punctate; with sparse, white setae interspersed with long, yellowish setae; apex of urosternite V emarginate.

*Legs.* Femora with sparse, white setae interspersed with long, yellowish setae. Tibiae with moderately abundant, long, yellowish setae (mainly towards apex), sparse, short, white setae at base.

*Dimensions in mm* (female). Total length, 6.20; length of prothorax at center, 1.20; anterior width of prothorax, 1.00; posterior width of prothorax, 1.10; humeral width, 1.65; elytral length, 4.50.

##### Type material.

Holotype female from BOLIVIA, *Santa Cruz*: Potrerillo del Guenda (Snake farm; 400m; 17°40'S / 63°27'W; 370–400 m), 14–16.X.2011, Skillman & Wappes col. (MNKM).

##### Remarks.

*Anobrium wappesi* differs from all other species in the genus by the absence of a tubercle at the lateral sides of the prothorax. It differs from *Anobrium fraterculum* Galileo & Martins, 2012 by the pronotum not microsculptured (pronotum distinctly microsculptured in *Anobrium fraterculum*). It can be distinguished from *Anobrium oberthueri* Belon, 1903 by the distinctly more abundant elytral pubescence (sparser in *Anobrium oberthueri*), dark-brown elytra, femora, and tibiae (reddish-brown in *Anobrium oberthueri*), and prothorax about as long as wide (distinctly longer than wide in *Anobrium oberthueri*).

Although the general appearance of *Anobrium wappesi* be more similar to that of *Anobrium fasciatum* Galileo & Martins, 2002, it can be included in the alternative of couplet “7”, from Galileo and Martins (2002) (translated; modified):

**Table:** 

7(6)	Vertex and pronotum without haloed punctures; center of pronotum without microsculpture. Bolivia, Brazil (Rondônia, Pará, Mato Grosso, Minas Gerais	***Anobrium oberthueri* Belon, 1903**
–	Vertex and pronotum with haloed punctures; center of pronotum microsculptured	**7**’
7’(7)	Lateral sides of prothorax with distinct tubercle. Brazil (São Paulo, Rio Grande do Sul)	***Anobrium fraterculum* Galileo & Martins, 2002**
–	Lateral sides of prothorax unarmed. Bolivia (Santa Cruz)	***Anobrium wappesi* sp. n.**

##### Note.

Belon (1903) described his species of *Anobrium* as *Anobrium oberthüri*. The specific epithet was emended to *Anobrium oberthuri* by Monné (1993). However, according to ICZN (1999: Article 32.5.2.1), the correct spelling of the name should be *Anobrium oberthueri*. Moreover, the species description has been cited as having been published in 1902. However, according to the Bulletin de la Société Entomologique de France, issue number 20 (“Dates d’apparition des numéros du Bulletin de 1902”), in which the species was described, was distributed on 22^nd^ January 1903.

### Acanthoderini Thomson, 1860

#### Cotycicuiara wappesi sp. n.

Taxon classificationAnimaliaColeopteraCerambycidae

http://zoobank.org/D9340A3F-9482-4824-9855-6B8CC6C3D454

Figs 7–9

##### Description.

*Holotype male.* Color. Integument dark-brown; about distal half of labrum and palpi reddish-brown.

*Head.* Frons, vertex and antennal tubercles finely, abundantly punctate; pubescence yellowish white (more whitish depending on angle of incidence of light), almost obliterating integument; with some long, dark setae close to lower eye lobes. Coronal suture distinct from clypeus to approximately middle of upper eye lobes. Area between eyes and antennal socket with narrow, dense, yellowish white band of pubescence. Area behind upper eye lobes finely, densely punctate; with dense band of yellowish white pubescence close to eyes, less dense towards prothoracic margin. Area behind lower eye lobes finely, abundantly punctate; with band of yellowish white pubescence close to eyes, enlarged towards gena, glabrous towards prothoracic margin. Genae with dense yellowish white pubescence, except for narrow distal band and area under lower eye lobes with sparse pubescence; with sparse, long, dark setae. Distance between upper eye lobes equal to 0.15 times length of scape; distance between lower eye lobes equal to 0.65 times length of scape. Antennae as long as 1.8 times elytral length; reaching elytral apex at apex of antennomere VIII; scape, pedicel and antennomeres III–VIII with yellowish white pubescence, not obliterating integument; antennal formula based on antennomere III: scape = 0.79; pedicel = 0.28; IV = 1.01; V = 0.82; VI = 0.73; VII = 0.66; VIII = 0.57; IX = 0.58; X = 0.54; XI = 0.47.

**Figures 7–12.**
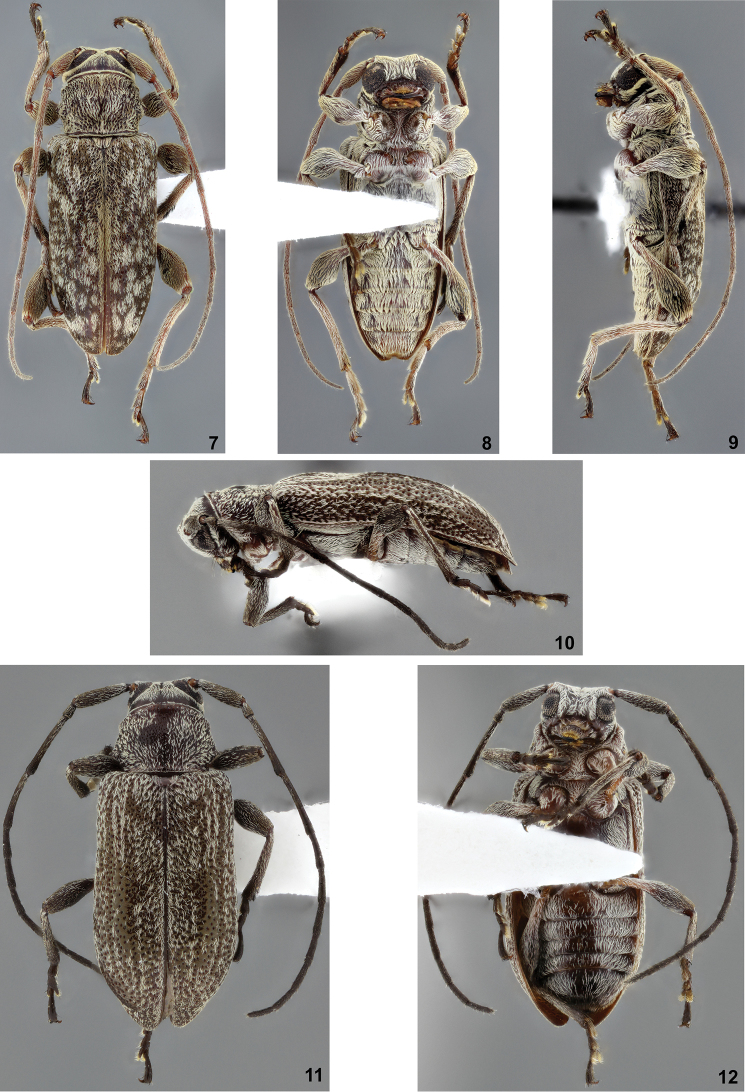
**7–9** *Cotycicuiara wappesi*, holotype male: **7** dorsal habitus **8** ventral habitus **9** lateral habitus **10–12** *Nesozineus wappesi*, holotype female: **10** lateral habitus **11** dorsal habitus **12** ventral habitus.

*Thorax.* Pronotum moderately coarsely, abundantly punctate; pubescence yellowish white, mixed with yellowish brown pubescence; with very sparse, long, dark setae near posterolateral angles. Lateral sides of prothorax with conical tubercle at about middle; pubescence (somewhat denser close to anterior margin) and sculpture as on pronotum. Ventral surface with yellowish white pubescence (more whitish depending on angle of incidence of light), not obliterating integument, denser in narrow band on metasternum close to mesocoxal cavities and apex of mesosternal process. Scutellum with yellowish white pubescence. Elytra. Moderately coarsely, abundantly punctate on basal third, gradually sparser towards apex; pubescence (Fig. 7) whitish, mixed with brownish pubescence; on basal two-thirds, moderately wide band of yellowish brown pubescence around suture; on each side of basal quarter, well-marked, slightly raised gibbosity, adjacent to transverse, slightly marked depression; elytral apex sub-truncate.

*Abdomen.* Urosternites with dense, yellowish white pubescence (more whitish depending on angle of incidence of light), almost obliterating integument. Legs. Femora and tibiae with yellowish white pubescence; length of metatarsomere V equal to 0.75 times II–III together.

*Dimensions in mm* (holotype/male). Total length, 8.40/8.35–8.50; length of prothorax at center, 1.75/1.55–1.60; anterior width of prothorax, 1.80/1.85–2.05; posterior width of prothorax, 1.95/1.95–1.95; largest width of prothorax, 2.4/2.20–2.50; humeral width, 2.95/3.00–3.10; elytral length, 6.10/6.20–6.20.

##### Type material.

Holotype male from BOLIVIA, *Santa Cruz*: Potrerillo del Guenda (Snake farm; 17° 40’ 15” S; 63° 27’ 26” W 400 m), 14-23-30.X.2013, Wappes & Kuckartz col. (MNKM). Paratypes – BOLIVIA, *Santa Cruz*: 83 km N Camiri (900 m, Road to Itai, 6–8 km E Highway 9; 19° 19’ S; 63°25'W), 2 males, 4–5.XII.2012, Skillman, Wappes & Bonaso col. (ACMT, MZSP).

##### Remarks.

*Cotycicuiara wappesi* differs from *Cotycicuiara acuminata* Galileo & Martins, 2012 as follows: elytral apex sub-truncate (distinctly acuminate in *Cotycicuiara acuminata*); pronotum abundantly punctate (mostly impunctate in *Cotycicuiara acuminata*); elytra with whitish pubescence occupying large areas (mostly yellowish brown in *Cotycicuiara acuminata*). It can be distinguished from *Cotycicuiara chionea* Martins & Galileo, 2010 by its humeral width, equal to approximately 1.2 times the largest width of head (approximately 1.7 times the largest width of the head in *Cotycicuiara chionea*), and elytral pubescence not forming notably dense spots (which occur in *Cotycicuiara chionea*).

*Cotycicuiara wappesi* can be included in the alternative of couplet “10”, from Martins and Galileo (2010):

**Table:** 

10’(9)	Elytral pubescence uniformly distributed on basal and distal half. Bolivia	***Cotycicuiara wappesi* sp. n.**
–	Elytral pubescence denser on basal half than on distal half	**10**
10(10’)	Pronotum with wide central longitudinal band of yellowish pubescence and sides with white pubescence; anterior half of elytra covered by white pubescence, except on contrasting punctures; posterior half with several spots of white pubescence. Brazil (Minas Gerais, Espírito Santo	***Cotycicuiara nivaria* Martins & Galileo, 2010**
–	Pronotum completely covered with white pubescence; elytra with a narrow white basal area, bordered posteriorly by a transverse less pubescent area, and with irregular white spots on the remaining surface. Brazil (Minas Gerais, Rio de Janeiro)	***Cotycicuiara chionea* Martins & Galileo, 2010**

#### Nesozineus wappesi sp. n.

Taxon classificationAnimaliaColeopteraCerambycidae

http://zoobank.org/B76DAACE-DFA4-4961-A1AC-19978DBBFD30

Figs 10–12

##### Description.

*Holotype female.* Color. Integument black; peduncle of femora partially dark-brown; pro- and mesosternal process brown; basal projection of urosternite I reddish-brown. General pubescence grayish-white.

*Head.* Frons, vertex and antennal tubercles microsculptured; pubescence abundant but not obliterating integument. Coronal suture distinct from clypeus to anterior edge of prothorax. Area behind eyes microsculptured; pubescence denser close to eyes than towards prothoracic margin. Genae microsculptured, sparsely pubescent. Distance between upper eye lobes equal to 0.35 times length of scape; distance between lower eye lobes, in frontal view, equal to 0.65 times length of scape. Antennae as long as 1.65 times elytral length; reaching elytral apex at middle of antennomere IX; scape, pedicel and basal half of antennomeres III–IV with sparse, grayish-white pubescence; antennomeres III–IV enlarged at distal inner side; antennal formula based on antennomere III: scape = 0.82; pedicel = 0.22; IV = 0.98; V = 0.52; VI = 0.50; VII = 0.47; VIII = 0.47; IX = 0.42; X = 0.45; XI = 0.50.

*Thorax.* Pronotum finely, abundantly punctate; pubescence moderately abundant, not obliterating integument; disc slightly convex, slightly depressed on each side of basal quarter. Sides of prothorax with distinct, conical tubercle at basal half; tumid close to lateroanterior angles; punctures closer than on pronotum; pubescence dense in wide band close to anterior margin, sparser towards posterior margin. Ventral side with moderately dense pubescence, not totally obliterating integument. Scutellum laterally pubescent, glabrous in wide central area. Elytra. Coarse, abundantly punctate; with wide, curve depression from near humerus to apex of middle third (deep on basal third), together on elytra forming X-like; discal sides at distal third tumid (not reaching apex), and the vertical lateral side somewhat depressed; apex moderately narrow, individually rounded; pubescence laterally sub-aligned in rows.

*Abdomen.* Urosternites laterally pubescent, centrally with pubescence distinctly sparser. Legs. Femora and tibiae with pubescence not dense; meso- and metafemoral club not notably enlarged.

*Dimensions in mm* (female). Total length, 4.85; length of prothorax at center, 1.00; anterior width of prothorax, 1.25; posterior width of prothorax, 1.25; largest width of prothorax, 1.55; humeral width, 1.75; elytral length, 3.55.

##### Type material.

Holotype female from BOLIVIA, *Santa Cruz*: Cordillera Province (Road to Itai, 83 km N Camiri; 19°20'S / 63°28'W; 890 m), 17–18.XII.2011, Wappes, Lingafelter and Woodley col. (MNKM).

##### Remarks.

*Nesozineus wappesi* differs from *Nesozineus unicolor* Martins et al., 2009 by its thicker dorsal pubescence, coarser and sparser elytral punctures, and elytral surface with depressed areas (uniform in *Nesozineus unicolor*). It differs from *Nesozineus lineolatus* Galileo & Martins, 1996 by its distinctly more robust body and the lateral tubercle of prothorax not spiny and shorter.

*Nesozineus wappesi* can be included in the alternative of couplet “4”, from Galileo and Martins (1996) (translated):

**Table:** 

4’(3)	Scape shorter than antennomere III; elytra as long as twice humeral width. Bolivia	***Nesozineus wappesi* sp. n.**
–	Scape as long as antennomere III; elytra longer than twice humeral width	**4**
4(3’)	Elytral pubescence uniformly distributed. Venezuela, Bolivia, Brazil (Maranhão, Piauí).	***Nesozineus apharus* Galileo & Martins, 1996**
–	Elytral pubescence more concentrated on four narrow, longitudinal rows at basal two-thirds. Brazil (Maranhão, Alagoas, Sergipe, Mato Grosso do Sul), Paraguay.	***Nesozineus lineolatus* Galileo & Martins, 1996**

#### Psapharochrus wappesi sp. n.

Taxon classificationAnimaliaColeopteraCerambycidae

http://zoobank.org/B9A396FE-B504-43A2-AAB6-F76E76AED3BA

Figs 13–15

##### Description.

*Holotype female.* Color. Integument dark-brown; base of gula yellowish brown; apex of last maxillary and labial palpomere yellowish; tibiae brown.

*Head.* Frons coarsely, sparsely punctate; pubescence dense, mostly yellowish brown, laterally more yellowish, obliterating integument. Antennal tubercles impunctate, with yellowish brown pubescence obliterating integument. Area between antennal tubercles and middle of upper eye lobes somewhat flat, coarsely, partially confluently punctate; pubescence centrally yellowish brown, laterally yellowish, not obliterating punctures. Vertex distinctly raised from middle of upper eye lobes; moderately finely, sparsely punctate; pubescence mostly yellowish, distinctly less dense around coronal suture and close to anterior edge of prothorax, with wide spot of yellowish brown pubescence on each side of coronal suture. Coronal suture distinct from clypeus to anterior edge of prothorax. Area behind upper eye lobes finely, rugose-punctate close to eyes, moderately abundantly punctate towards prothorax; with band of yellowish pubescence, not totally obliterating integument, gradually narrowed towards lower eye lobes. Area behind lower eye lobes fine, abundantly punctate, gradually sparser towards gula; with narrow band of yellowish pubescence close to eyes, almost glabrous on the remaining surface. Gula with yellowish pubescence, not obliterating integument. Submentum with short, yellowish pubescence on area near mentum. Distance between upper eye lobes equal to 0.45 times length of scape; distance between lower eye lobes, in frontal view, equal to length of scape. Antennae as long as 1.65 times elytral length; reaching elytral apex at basal quarter of antennomere IX; antennomere III with ring with whitish pubescence on base, followed by wide ring with brown-yellowish pubescence, central ring with whitish pubescence mixed with brown-yellowish pubescence, with wide ring with dark-brown pubescence, and narrow apical band of whitish pubescence; antennomeres IV–XI with whitish pubescence on basal half, and dark-brown pubescence on distal half; antennal formula based on antennomere III: scape = 0.65; pedicel = 0.23; IV = 0.73; V = 0.58; VI = 0.48; VII = 0.45; VIII = 0.43; IX = 0.35; X = 0.27; XI = 0.25.

**Figures 13–15.**
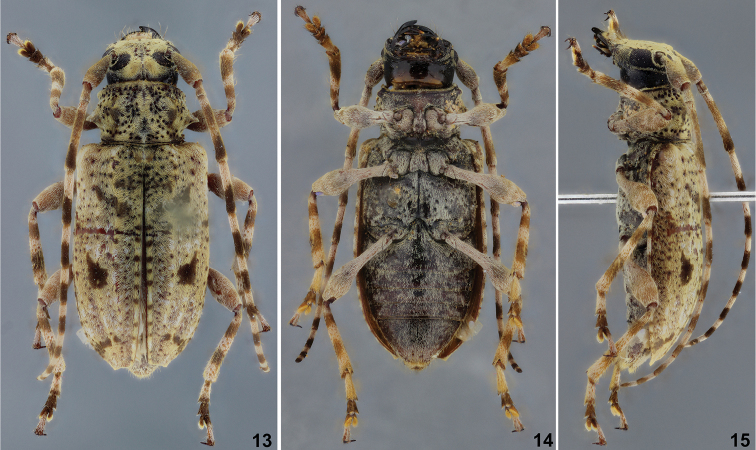
*Psapharochrus wappesi*, holotype female: **13** dorsal habitus **14** ventral habitus **15** lateral habitus.

*Thorax.* Lateral tubercles of prothorax large, conical, apex slightly directed upwards and backwards. Lateral tubercles on pronotal disc large, raised, apex not acute; central tubercle carina-like, from basal quarter to near apex; surface among tubercles coarsely, deeply, moderately abundantly punctate; central pubescence yellowish between lateral tubercles; with weakly defined band of yellowish brown pubescence crossing lateral tubercles, from base to apex; laterally with yellowish pubescence. Lateral sides of prothorax coarsely, deepply, moderately abundantly punctate; pubescence as on lateral of pronotum. Prosternum with short, not dense (laterally slightly denser), pale yellow pubescence (more whitish depending on angle of incidence of light). Prosternal process with lateral sides raised about middle; pubescence as on center of prosternum, slightly denser on transverse band in middle and laterally on distal half. Mesosternum and mesosternal process with pubescence slightly denser than on center of prosternum (shorter, sparser on basal center of mesosternum). Metasternum coarsely, deeply, sparsely punctate; pubescence as on mesosternal process. Scutellum with yellowish pubescence in center, yellowish brown on sides. Elytra. Moderately coarsely, sparsely punctate; each elytron with two distinct carinae from near base to near apex; pubescence (Fig. 13) mostly yellowish; with oblique band with brown pubescence on basal third, not reaching lateral side and suture; large spot of brown pubescence immediately adjacent to center; on distal quarter, oblique band with brown pubescence, starting at lateral margin, not reaching suture; close to suture, on distal two-thirds, small spots of brown pubescence, smaller, sparser towards apex; apex slightly obliquely truncate, with outer angle projected.

*Abdomen.* Pubescence pale yellow (more whitish depending on angle of incidence of light), not obliterating integument. Legs. Tibiae with yellowish pubescence, with wide ring of brownish pubescence on each half. Tarsomere I with yellowish pubescence, except for narrow apical band with dark-brown pubescence; tarsomeres II–IV with dark-brown pubescence; tarsomere V with yellowish pubescence, sparser near apex.

*Dimensions in mm* (female). Total length, 11.5; length of prothorax at center, 2.1; anterior width of prothorax, 2.9; posterior width of prothorax, 2.7; largest width of prothorax, 3.7; humeral width, 4.2; elytral length, 7.6.

##### Type material.

Holotype female from BOLIVIA, *Tarija*: 5 km W of Villamontes (Foothill Chaco Forest; 21°17'S / 63°28'W; *ca.* 500 m), 13.I.2008, R. Clarke & S. Zamalloa col. (MNKM).

##### Remarks.

*Psapharochrus wappesi* differs from *Psapharochrus piraiuba* Martins & Galileo, 2003 as follows: elytra with distinct spots of brown pubescence (absent in *Psapharochrus piraiuba*); pronotal punctures sparser (denser in *Psapharochrus piraiuba*); antennae in female surpassing the elytral apex (not reaching the elytral apex in *Psapharochrus piraiuba*); and tibiae with distinct rings of pubescence (not present in *Psapharochrus piraiuba*). *Psapharochrus wappesi* differs from *Psapharochrus ridleyi* (Waterhouse, 1894) by the sub-parallel elytral margins from their base to the base of their distal third (uniformly narrowed from base to apex in *Psapharochrus ridleyi*), antennae in female distinctly surpassing the elytral apex (at most, slightly surpassing elytral apex in *Psapharochrus ridleyi*), and flatter body (distinctly convex in *Psapharochrus ridleyi*).

## Supplementary Material

XML Treatment for Xenofrea wappesi

XML Treatment for Anobrium wappesi

XML Treatment for Cotycicuiara wappesi

XML Treatment for Nesozineus wappesi

XML Treatment for Psapharochrus wappesi
